# Epidemiological trends and determinants of mumps outbreaks: a systematic review and meta-analysis

**DOI:** 10.3389/fpubh.2025.1711759

**Published:** 2025-12-04

**Authors:** Ritik Agrawal, Tanveer Rehman, Deepti Sinha, Poulomee Chakraborty, Manikandanesan Sakthivel, Dewesh Kumar, Srijeeta Mitra, Afeeq Karumathil, Srikanta Kanungo, Sanghamitra Pati

**Affiliations:** 1 ICMR Regional Medical Research Centre, Bhubaneswar, Odisha, India; 2 Model Rural Health Research Unit, Ranchi, Jharkhand, India; 3 ICMR-National Institute of Epidemiology, Chennai, Tamil Nadu, India; 4 Rajendra Institute of Medical Sciences, Ranchi, Jharkhand, India

**Keywords:** attack rate, MMR vaccine, mumps, outbreak, vaccination

## Abstract

**Background:**

Despite widespread vaccination programs, mumps has resurged globally. This systematic review and meta-analysis assessed the epidemiological characteristics, attack rates (ARs), and complications of mumps outbreaks worldwide from 2004 to 2024.

**Methods:**

We systematically searched MEDLINE, Embase, CINAHL and Google Scholar for studies reporting on mumps outbreaks. Confirmed mumps cases, defined by WHO criteria, were included across all age groups. Epidemiological characteristics were summarized using the Time-Place-Person format. Pooled ARs and complication rates were calculated using random-effects models. Subgroup analyses examined variations by age, region, vaccination status, and outbreak period. A random-effects meta-regression and leave one out sensitivity analysis was used to explore the influence of study-level characteristics heterogeneity in the attack rate among mumps outbreak studies. Heterogeneity was assessed using Cochran’s Q and I^2^, and publication bias was evaluated with funnel plots and Egger’s test.

**Results:**

A total of 47 studies from 21 countries reporting 71,174 mumps cases, were included in the systematic review, with 30 studies in the meta-analysis. The pooled AR of mumps outbreaks was 14.5% (95% CI: 12.91–16.11), with adults having the highest AR (31.8%). The pooled complication rate was 10.3% (95% CI: 5.7–14.9), with orchitis being the most common complication (63.1%). Temporal trends showed peaks during 2004–2009 and 2016–2020, while regional analysis revealed higher ARs in the Americas (29.2%) and Eastern Mediterranean (28.8%) regions compared to Europe (7.6%) and South-East Asia (9.6%). Among vaccinated individuals, ARs were highest with a single dose (35.7%) and lowest with three doses (10.1%).

**Conclusion:**

Mumps outbreaks remain a global concern due to waning vaccine-induced immunity. Incorporating a third MMR booster dose into vaccination schedules is recommended, particularly for high-risk groups, to reduce ARs and complications effectively.

**Systematic review registration:**

https://www.crd.york.ac.uk/PROSPERO/.

## Introduction

Mumps, a highly contagious viral illness caused by a member of the *Paramyxoviridae* family, spreads primarily through respiratory droplets (1). Clinically, it is characterised by inflammation and swelling of the parotid glands. However, complications such as meningitis, orchitis, encephalitis, pancreatitis, nephritis, and, in rare cases, mortality significantly contribute to the disease’s public health burden, highlighting the critical importance of prevention and control strategies (2, 3). The advent of widespread vaccination programs, particularly with the Measles, Mumps, and Rubella (MMR) vaccine in the 1970s, has dramatically reduced the global mumps incidence worldwide (4). Mumps vaccination was first introduced in high-resource settings (e.g., the U. S., Canada, and Europe), but its global rollout has been uneven and World Health Organization (WHO) data indicates that as of 2023, numerous low- and middle-income countries (LMICs) such as India, Lao People Democratic Republic, and most African nations still lack routine mumps immunization, highlighting persistent inequities in vaccine access (5, 6). Despite this progress, mumps continues to pose significant global health challenges (7, 8). The attack rate (AR), defined as the proportion of individuals who develop the disease among those exposed during an outbreak, is a critical metric for understanding the dynamics of mumps outbreaks (9). The WHO estimates approximately 500,000 mumps cases are reported annually worldwide, with fluctuations in incidence reflecting its complex epidemiological landscape (7, 8). In 2023, WHO reported 384,785 mumps cases globally, a 1% increase from the previous year (10). Recent resurgences, such as those observed in Europe between 2021 and 2022, particularly among individuals aged 10 years and older, suggest shifting patterns of transmission and immunity (11). Moreover, mumps outbreaks impose a substantial economic burden; a U. S. study estimated a per-case cost of $9,459, including direct medical expenses and productivity losses (12). In LMICs, endemic mumps circulation remains a persistent challenge due to disparities in vaccination coverage and healthcare access, underscoring the urgent need for adaptive vaccination strategies and robust surveillance systems (13).

Several key factors influence mumps outbreaks, including vaccination coverage, timing of vaccination, waning immunity, viral strain variability, and demographic shifts (14). These determinants play pivotal roles in outbreak dynamics, affecting population vulnerabilities, predicting future outbreaks, and guiding the implementation of control measures (15, 16). Notably, recent outbreaks have disproportionately affected adolescents and young adults, marking a transition from the traditional burden among younger children (13, 17). For example, in Spain from 1998 to 2003 (Period 1, P1), children aged 1–4 years experienced the highest incidence rate (71.7 cases per 100,000 population). Over subsequent periods, this pattern shifted towards adolescents and young adults, with higher incidence rate ratios observed among those aged 15–24 years (P2 = 1.46; P3 = 2.68) and adults aged 25–34 years (P2 = 2.17; P3 = 4.05) during 2004–2009 (P2) and 2010–2014 (P3) (18). These trends are compounded by behavioural and societal challenges such as vaccine hesitancy, inconsistent immunization adherence, and strained public health resources (19, 20). Understanding these shifts is critical for developing targeted vaccination strategies to address the evolving dynamics of mumps transmission.

Vaccination remains the cornerstone of mumps prevention. Two-dose vaccine schedules offer higher protection (vaccine effectiveness, VE: 64.0–92.4%) compared to single-dose regimens (VE: 47.4–86.0%) (21, 22). Vaccination has reduced mumps incidence by 66–88% in high-risk areas (23, 24). However, waning immunity, particularly among individuals vaccinated over a decade ago, presents a significant challenge (25–27). Individuals vaccinated 13 years prior are nine times more likely to contract mumps than those recently vaccinated (19, 20). To address waning immunity, the Centres for Disease Control and Prevention (CDC) recommend administering a third dose of MMR vaccine during outbreaks for individuals previously vaccinated with two doses. This approach aims to bolster immunity among high-risk groups and mitigate outbreak severity (19, 28, 29).

Measuring AR globally and regionally provides invaluable insights into the effectiveness of vaccination programs, susceptibility patterns, and outbreak severity across populations (30). Such analyses help identify immunity gaps, emerging risk factors, and the role of waning immunity in propagating outbreaks. Additionally, AR quantifies the public health impact of outbreaks, aiding resource allocation and outbreak response planning (31). Recent estimates show ARs ranging from 5.6 to 13.2% even in vaccinated communities, highlighting the complex waning vaccine efficacy, shifting epidemiological trends, and evolving viral strains underscoring the pressing need for ongoing research (15, 32–35). Given these challenges, it is imperative to generate robust evidence regarding the shifting epidemiology of mumps. Despite these concerns, synthesized global evidence on ARs, booster dose efficacy, and complications remains limited. In this context, we aimed to determine epidemiological characteristics and determinants of mumps outbreaks globally by assessing global and regional ARs, identifying key outbreak determinants, and evaluating complication rates associated with mumps.

## Methods

### Protocol and registration

We conducted a systematic review and meta-analysis of cross-sectional, cohort and case–control studies reporting on the epidemiological characteristics of mumps outbreaks. This review was registered with PROPSPERO (CRD42024572629) (36). The study design, selection, screening, analysis, and reporting adhered to the latest PRISMA-2020 guidelines (37), with the detailed PRISMA checklist provided in Supplementary Table 1.

### Information source and search strategy

A systematic literature search was initially conducted on July 30, 2024, and updated on August 19, 2024. We searched peer-reviewed databases, including MEDLINE (via PubMed), Embase (via Ovid), and CINAHL (via EBSCO), along with Google Scholar to capture relevant grey literature including the studies published between 2004 and 2024. A predefined search strategy combined text words and Medical Subject Headings (MeSH) terms. For example, in PubMed, we searched for *Mumps* (MeSH) combined with title and abstract searches for “Epidemic parotitis,” “Mumps viruses,” and “Myxovirus parotitis.” Then, we searched with the MeSH term *Disease Outbreaks,* combined with a search of title and abstract using “Disease hotspot,” “Outbreaks,” and “Epidemics.” The search was restricted to studies published in English. Reference lists of included studies were manually screened by two independent reviewers (RA, SM) to identify additional relevant articles. Systematic review experts (MS, TR) assisted in refining the search strategy. The detailed search methodology is outlined in Supplementary Table 2.

### Eligibility criteria

We defined the inclusion criteria using the CoCoPop framework (38).

Condition: Confirmed mumps cases, as defined by WHO.

Context: Cases reported during an outbreak.

Population: Global populations of any age group.

We defined mumps cases according to WHO clinical and laboratory criteria (39). Suspected cases were identified by acute onset of unilateral or bilateral parotid gland swelling lasting >2 days without an alternative cause. Confirmed cases included laboratory-confirmed results (e.g., mumps-specific IgM antibodies, mumps virus RNA detected via RT-PCR, or virus isolation) or cases linked to a known outbreak.

Eligible studies met the following criteria:

- Reported a mumps outbreak in the last two decades (between 2004 and 2024).
- Included an epidemiological curve.
- Specified the outbreak duration (short or extended).

The exclusion criteria were as follows:

- Not original research (e.g., reviews, abstracts, editorials, commentaries).
- Lacked methodological details or diagnostic confirmation.
- Focused on diseases other than mumps or co-infections without specific mumps data. Potential confounders, such as misdiagnoses of parainfluenza or Epstein–Barr virus, were excluded (40–42).

### Study selection

We identified, extracted, and compiled the eligible studies into CSV and RIS formats. Preliminary deduplication and screening were conducted in Rayyan software (43). The selection process included:

1. Primary screening: Two independent reviewers (DS, PC) screened titles, abstracts and keywords (inter-rater agreement: 85–94%). Disagreements were resolved through discussion or consultation with a third reviewer (RA). Full-text retrieval was based on eligibility criteria.
2. Secondary screening: Full-text articles were reviewed by the same reviewers (inter-rater agreement: 94–95%).
3. Final selection: Inclusion decisions were finalized through consensus among investigators (TR, MS).

### Data collection and data items

We extracted data using a pre-designed Excel spreadsheet (by DS and PC) and cross-checked it (by RA, TR, and MS). Disagreements were resolved through discussion. Missing data were requested from corresponding authors until September 21, 2024. Extracted data included:

- Study Characteristics: Authors, year, and country.
- Methodological Details: Study setting, design, population demographics (age, gender).
- Outbreak Information: Duration, timeline, diagnostic methods, case definitions, confirmed cases, population at risk.
- Clinical Features: Symptoms (e.g., fever, gland swelling), complications (e.g., orchitis, meningitis, encephalitis).
- Vaccination Data: Status of unvaccinated individuals and those with one, two, or three doses.

### Data analysis

We analyzed data using R (V.3.0.3, The R Foundation for Statistical Computing, Vienna, Austria) (44). Epidemiological characteristics were summarized using the Time-Place-Person (TPP) format (45, 46). Descriptive statistics included proportions, means with standard deviations (SD), and medians with interquartile ranges (IQR), depending on data distribution. Temporal trends were analysed by grouping studies into three intervals: 2004–2009, 2010–2015, and 2016–2020, ensuring even distribution. While our search extended to studies published up to 2024, we found no published evidence of outbreaks beyond 2020 in the available literature. Geographic distribution was analysed using the WHO’s regional classification system (47). A choropleth map was generated in QGIS software to visually depict regional AR trends, highlighting variations across different WHO regions (48). Subgroup analyses were conducted to examine patterns of mumps complications. We estimated the pooled ARs and complication rate of mumps globally. The AR was calculated for each study using the reported number of mumps cases (numerator) divided by the total at-risk population (denominator). The complication rate was computed as the proportion of mumps cases that developed any of the specific complications: orchitis, meningitis, encephalitis, pancreatitis, oophoritis, or hearing loss expressed as a percentage of total cases. We assessed statistical heterogeneity across studies using Cochran’s Q test (with *p* < 0.10 indicating significant heterogeneity) and the I^2^ statistic, interpreting I^2^ values as follows: 0–40% as negligible, 30–60% as moderate, 50–90% as substantial, and 75–100% as considerable heterogeneity (49–51). Based on the degree of heterogeneity, a random-effects model was applied when heterogeneity was significant, while a fixed-effects model was used otherwise. Forest plots were created to visually summarize pooled estimates of ARs and complication rates, along with their confidence intervals (CIs). Sensitivity analyses, including “leave-one-out” methods, were performed to assess the robustness of pooled estimates by sequentially removing individual studies and observing their impact (52). Additionally, subgroup analyses explored AR determinants by age groups, WHO regional classifications, vaccination statuses, and years of outbreak. This approach accounted for potential geopolitical and socioeconomic differences influencing outbreak dynamics. A random-effects meta-regression with restricted maximum likelihood (REML) method was used to explore the influence of study-level characteristics heterogeneity in the attack rate among mumps outbreak studies (53). Different packages were used in R for the data analysis such as the meta-analyses of proportions were performed using the meta package (metaprop), while meta-regression and random-effects models were conducted with the metafor package (rma, rma.glmm). Forest plots and funnel plots were generated using a combination of meta, metafor with diagnostic checks supported by dmetar. Publication bias was assessed through visual inspection of funnel plots, and Egger’s regression test was used for quantitative evaluation, with *p* ≤ 0.05 considered statistically significant (54).

### Risk of bias assessment

Three reviewers (DS, PC, and RA) independently assessed bias using Joanna Briggs Institute (JBI) tools for cross-sectional, cohort, and case–control studies (55–57). Criteria included sample appropriateness, research aim clarity, and outcome accuracy. Scores (Yes = 1, No = 0, Unclear = 0.5) provided objective insights, helping us to determine the reliability of the findings and the potential for bias.

## Results

**Figure 1:**
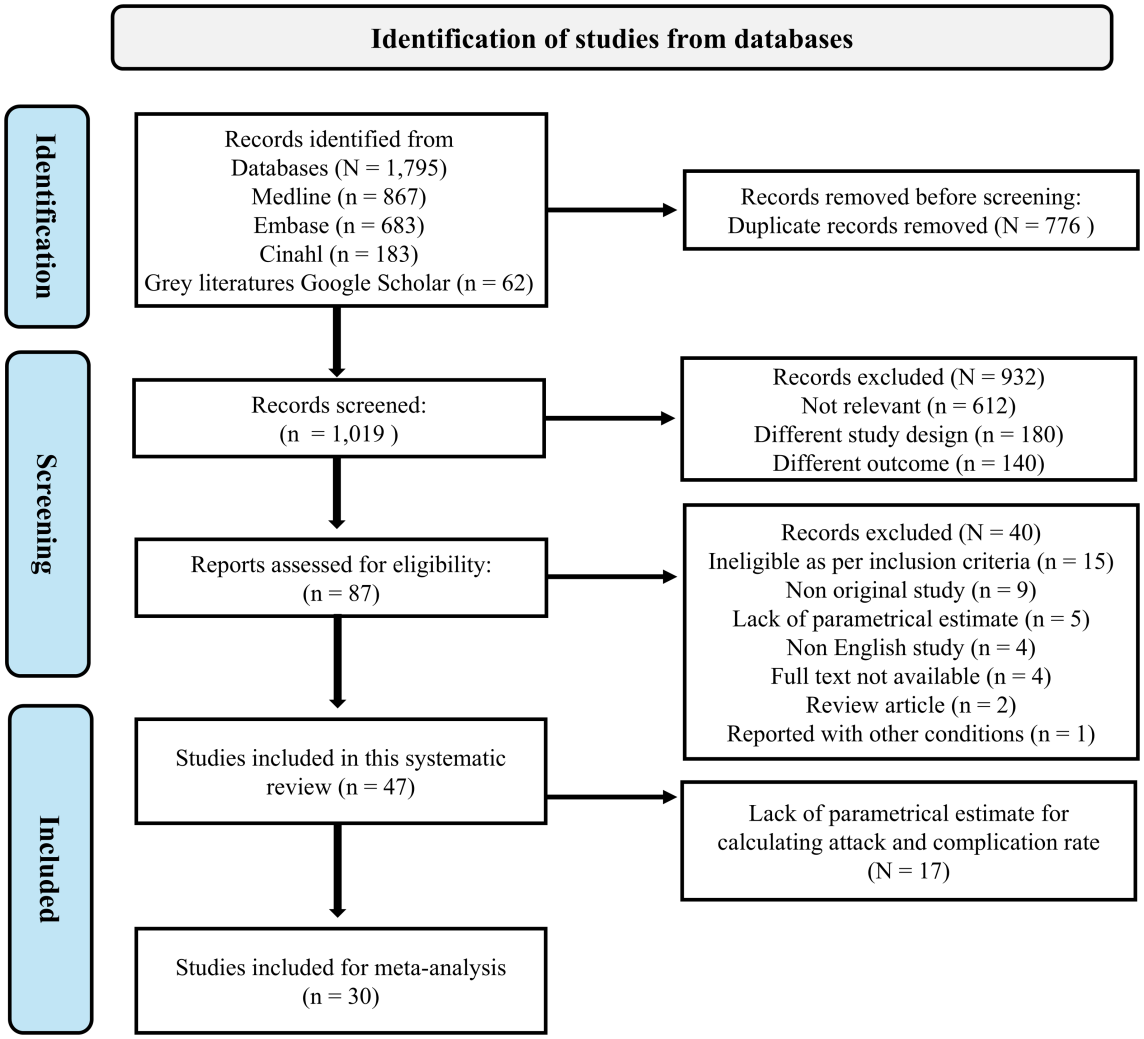
PRISMA flow of studies included in systematic review and meta-analysis.

We identified a total of 1,795 articles published between 2004 and 2024 through a systematic search. After applying the eligibility criteria, 87 articles were deemed potentially relevant, and 47 studies were included in the systematic review. Of these, 30 studies met the criteria for inclusion in the meta-analysis, while 17 studies were excluded due to insufficient parametric estimates for calculating ARs and complication rates. The details of all the studies identified with their reason for exclusion are provided in the Supplementary Table 3. The study selection process is illustrated in the PRISMA flow diagram (Figure 1).

### General characteristics of the included studies

The 47 studies represented data from 21 countries, documenting 71,174 cumulative mumps cases across 61 outbreaks between 2004 and 2024. The affected population ranged from 0 to 90 years of age (12, 16, 34, 58–101). Most studies reported a single outbreak whereas a few reported multiple outbreaks (62, 68, 74, 77, 83, 96). Among the cases, three fatalities were reported in a study from the Lao People’s Democratic Republic by Hubschen et al. (74). Study designs predominantly included cross-sectional studies, with two cohort studies (51, 70) and two case–control studies (49, 68). Diagnostic approaches varied, with 31 studies (65.9%) using a combination of laboratory criteria for mumps confirmation. The included studies are summarized in Table 1. The detailed template for data extracted from the included studies are provided in Supplementary Table 4.

**Table 1:** Summary characteristics of individual studies included in a systematic review of a mumps outbreak globally.

S. N.	Author name	Country	Study design	Length of Study period	Mumps cases	Reported outbreak duration (in months)	Age group	Attack rate (in percentage)	Vaccinations status
1	Walker et al. (58)	Australia	Case control study	October 2017 to October 2018	93	12	0–54	NA	Vaccinated and unvaccinated
2	Maillet et al. (59)	France	Cross sectional study	February to June 2013	62	5	18–25	1.20%	Vaccinated
3	Hukic et al. (60)	Bosnia	Cohort study	December 2010 to September 2012	7,895	21	1–64	19.70%	Vaccinated and unvaccinated
4	Tan et al. (61)	Canada	Observational study	February to October 2008	180	8	NA	47.22%	Vaccinated and unvaccinated
5	Schulte et al. (62)	United States	Cross sectional study	December 2018 to August 2019	102	10	20–57	47.06%	Vaccinated and unvaccinated
6	Waugh et al. (63)	Scotland	Observational study	October 2017 to May 2018	324	7	0–73	NA	Vaccinated and unvaccinated
7	Tilavat et al. (64)	India	Cross sectional study	May to June 2016	13	7	15–40	5.10%	Unvaccinated
8	Shah et al. (65)	Netherlands	Cross sectional study	October 2018 to March 2020	102	6	3–71	4.50%	Vaccinated and unvaccinated
9	Albertson et al. (66)	United States	Cross sectional study	April 2015 to May 2016	317	13	16–55	3.90%	Vaccinated and unvaccinated
10	Boxall et al. (67)	Czech Republic	Cross sectional study	1 January 2005 to 30 June 2006	5,998	18	0–80	NA	Vaccinated and unvaccinated
11	Gouma et al. (68)	Netherlands	Cross sectional study	1 September 2009 to 31 August 2012	822	36	18–22	NA	Vaccinated and unvaccinated
12	Cordeiro et al. (69)	Portugal	Cross sectional study	21 October 2012 to 14 March 2013	148	6	2–62	11.63%	Vaccinated and unvaccinated
13	Vaidya et al. (70)	India	Cross sectional study	2 October 2016 to 19 March 2017	139	6	17–42	28.06%	NA
14	Marx et al. (71)	United States	Cross sectional study	1 November 2016 to 28 March 2017	47	5	0–44	36.17%	Unvaccinated
15	Aasheim et al. (72)	United Kingdom	Cross sectional study	1 January to 13 April 2013	28	4	10–19	3.94%	Vaccinated and unvaccinated
16	Zamir et al. (73)	Israel	Descriptive cross-sectional study	6 September 2009 to 12 August, 2011	3,130	23	1–30	10%	Vaccinated and unvaccinated
17	Hubschen et al. (74)	Lao People’s Democratic Republic	Cross sectional study	10 March 2011 to 4 May 2013	118	3.6	1–45	4.96%	Unvaccinated
18	Ferenczi et al. (75)	Ireland	Cross sectional study	18 August 2018 to 24 January 2020	3,736	17.5	0–45	NA	Vaccinated and unvaccinated
19	Qin et al. (76)	China	Case control study	1 October 2016 to 31 January 2017	97	4	6–15	8.20%	Vaccinated and unvaccinated
20	Creed et al. (77)	Canada	Cross sectional study	1 May 2005 to 30 January, 2006	32	9	13–27	NA	Vaccinated
21	Brockhoff et al. (30)	Netherlands	Cohort study	1 September to 1 December, 2004	1,561	3	16–66	12.52%	Vaccinated and unvaccinated
22	Kutty et al. (78)	United States	Cross sectional study	24 September 2009 to 15 June 2010	2,503	4	0–24	12.78%	Vaccinated
23	Paul et al. (79)	India	Cross sectional study	14 August to 31 December, 2014	94	4	2–40	9.94%	Unvaccinated
24	Cohen et al. (80)	United Kingdom	Cross sectional study	1 January 2004 to 31 March 2005	311	15	2–12	NA	Vaccinated and unvaccinated
25	Jones et al. (81)	Australia	Cross sectional study	1 July 2007 to 30 June 2008	153	12	0–40	NA	Vaccinated and unvaccinated
26	Moghe et al. (82)	India	Cross sectional study	23 June 2016 to 10 September 2016	162	2.6	0.6–32	2.16%	Unvaccinated
27	Orlikova et al. (83)	Czech Republic	Cross sectional study	1 January 2007 to 31 December 2012	9,663	72	0–90	1.60%	Vaccinated and unvaccinated
28	Schmid et al. (84)	Austria	Cross sectional study	1 May to 28 August 2006	214	4	6–69	NA	Vaccinated and unvaccinated
29	Whelan et al. (85)	Netherlands	Cross sectional study	1 December 2009 to 200 April 2010	172	4	4–46	3.95%	Vaccinated and unvaccinated
30	Walker et al. (86)	United Kingdom	Cross sectional study	29 November 2010 to 31 January 2011	119	3	4–71	1.49%	Vaccinated and unvaccinated
31	Rajcevic et al. (87)	Serbia	Cross sectional study	16 January 2012 to 30 April 2012	119	4	3–37	NA	Vaccinated and unvaccinated
32	Zamir et al. (88)	Israel	Cross sectional study	7 September to 7 December 2009	173	3	1–25	9.60%	Vaccinated and unvaccinated
33	Bernard et al. (89)	Republic of Moldova	Cross sectional study	17 December 2007 to 2 March 2008	14,729	6	15–24	1.72%	Vaccinated and unvaccinated
34	Hukic et al. (90)	Bosnia and Herzegovina	Cross sectional study	1 December 2010 to 31 July 2011	5,261	8	15–29	NA	Vaccinated and unvaccinated
35	Raut et al. (91)	India	Cross sectional study	12 January to 1 February, 2014	31	1	4–13	8.52%	NA
36	Indenbaum et al. (16)	Israel	Cross sectional study	1 January to 28 August 2017	262	8	10–24	83.20%	Vaccinated and unvaccinated
37	Mossong et al. (92)	Luxembourg	Cross sectional study	18 August to 28 December, 2008	225	5	15–34	59.56%	Vaccinated and unvaccinated
38	Patel et al. (93)	United States	Cross sectional study	12 January to 30 April 2014	56	4	18–37	50.0%	Vaccinated and unvaccinated
39	Sane et al. (94)	Netherlands	Cross sectional study	1 September 2009 to 31 August 2012	1,557	36	18–25	NA	Vaccinated and unvaccinated
40	Anis et al. (12)	Israel	Cross sectional study	1 September 2009 to 31August 2010	5,239	12	10–19	13.44%	Vaccinated
41	Walkty et al. (95)	Canada	Cross sectional study	12 January to 5 February 2009	322	1	28–34	1.55%	Vaccinated and unvaccinated
42	Saboui et al. (96)	Canada	Cross sectional study	1 January 2016 to 31 July 2018	881	29	15–39	NA	Vaccinated and unvaccinated
43	McKay et al. (97)	Federated states of Micronesia	Cross sectional study	5 August to 1 November 2017	23	5	1–26	NA	Vaccinated and unvaccinated
44	Tiffany et al. (98)	United States	Cross sectional study	1 May 2017 to 31 July 2018	391	15	0.2–79	NA	Vaccinated and unvaccinated
45	Nedeljkovic et al. (99)	Serbia	Cross sectional study	16 January to 30 June 2012	335	15	4–58	NA	Vaccinated and unvaccinated
46	Golwalker et al. (100)	United States	Cross sectional study	1 January 2016 to 5 August 2017	281	21	0.2–82	NA	Vaccinated
47	Fields et al. (101)	United States	Cross sectional study	5 August 2016 to 5 August, 2017	2,954	12	5–17	41.63%	Vaccinated and unvaccinated

### Mumps vaccination policies across countries

Table 2 summarizes the mumps vaccination policies of countries included in the systematic review and meta-analysis. It details the year of mumps vaccine introduction, the number of doses in the national immunization schedule, the recommended ages for the first and second doses, and notes on booster policies. This information provides context for understanding the variability in mumps outbreak dynamics and the potential impact of booster doses.

**Table 2:** Mumps vaccination policies across countries included in this systematic review and meta-analysis as per WHO immunization schedule (6).

Country included in this study	Year of vaccine introduction	Dose in the national schedule	Age at 1st dose	Age at 2nd dose	Recommendations for booster dose
Australia	1994	2	12 months	18 months	3rd dose considered during outbreaks for individuals at increased risk
Austria	1995	2	9 months	Before school entry	No routine booster; catch-up campaigns during outbreaks
Bosnia and Herzegovina	1995	2	1 year	6 years	No routine booster; catch-up campaigns during outbreaks
Canada	1983	2	12–15 months	18 months – 6 years	No routine booster; catch-up campaigns during outbreaks
China	2007	2	8 months	18 months	Catch-up campaigns post-2008
Czech Republic	1993	2	13 months	5 years	No routine booster; catch-up campaigns during outbreaks
Federated States of Micronesia	1998	2	12 months	≥ 13 months	No routine booster; catch-up campaigns during outbreaks
France	1995	2	12 months	18 months	No routine booster; catch-up campaigns during outbreaks
India	Not in national program	0–2	Variable	Variable	Not included in national immunization program; private sector availability
Ireland	1995	2	12 months	4–5 years	No routine booster; catch-up campaigns during outbreaks
Israel	1995	2	12 months	6 years	3rd dose considered during outbreaks for individuals at increased risk
Lao People’s Democratic Republic	Not in national program	0	Not applicable	Not applicable	Not included in national immunization program
Luxembourg	1986	2	12 months	15–23 months	No routine booster; catch-up campaigns during outbreaks
Netherlands	1995	2	14 months	9 years	No routine booster; catch-up campaigns during outbreaks
Portugal	1995	2	12 months	5 years	No routine booster; catch-up campaigns during outbreaks
Republic of Moldova	1995	3	12 months	6–7 years	Recently they have recommended the 3^rd^ dose to be administered at 15 years of age.
Scotland	1988	2	12–13 months	3 years 4 months	No routine booster; catch-up campaigns during outbreaks
Serbia	2002	2	12–15 months	7 years	No routine booster; catch-up campaigns during outbreaks
United Kingdom	1988	2	12–13 months	3 years 4 months	3rd dose considered during outbreaks for individuals at increased risk
United States	1969	2	12 months	4 years	3rd dose considered during outbreaks for individuals at increased risk

### Epidemiological characteristics of mumps outbreaks

#### Time

Outbreaks occurred consistently over the 20-year study period, with peaks during 2004–2009 (38.3%, *n* = 18) and 2016–2020 (34.0%, *n* = 16), while fewer outbreaks were documented between 2010 and 2015 (27.7%, *n* = 13). The mean (SD) outbreak duration was 10.95 (12.42) months. These figures represent the distribution of published studies reporting outbreaks, and not surveillance-based incidence data; hence, they should be interpreted descriptively rather than as statistically significant temporal trends.

#### Place

The geographical distribution of outbreaks revealed distinct patterns across WHO regions:

- European Region (EUR): Represented 44.7% (*n* = 21) of outbreaks, with the highest concentration in the Netherlands (5 studies, 10.6%) (34, 65, 68, 85, 94), and the United Kingdom (3 studies, 6.4%) (72, 80, 86).
- Region of the Americas (AMR): Accounted for 25.5% (*n* = 12) of outbreaks, primarily in the United States (8 studies, 17.0%) (62, 66, 71, 78, 93, 98, 100, 101), and Canada (4 studies, 8.5%) (61, 77, 95, 96).
- South-East Asia Region (SEAR): Represented 12.8% (*n* = 6), with outbreaks in India (5 studies, 10.6%) (64, 70, 79, 82, 91), and Lao People’s Democratic Republic (1 study, 2.1%) (72).
- Western Pacific Region (WPR): Comprised 8.5% (*n* = 4), with outbreaks in Australia (2 studies, 4.3%) (58, 81), China (76), and the Federated States of Micronesia (97).
- Eastern Mediterranean Region (EMR): Contributed 8.5% (*n* = 4), with all outbreaks reported in Israel (12, 16, 73, 88).
- Africa Region (AFR): No outbreaks were reported from this region.

#### Person

Demographic and clinical characteristics of outbreaks were as follows:

- Age Distribution: Children aged 0–10 years accounted for 66.7% (32 studies) of cases, followed by adolescents aged 11–18 years (25.0%, 12 studies), often linked to university or workplace outbreaks. Adults (≥19 years) accounted for 8.3% (3 studies) of cases.
- Gender: Most studies (91.5%) included mixed-gender populations. However, males represented 57% (*n* = 40,811) of cases, compared to females (38%, *n* = 27,254).
- Clinical Features: Parotitis was the most frequently reported symptom, accompanied by fever, cold, and cough in some cases. Rare symptoms included difficulty swallowing (64, 79, 82) and earache (64, 79, 88, 100). Complications such as orchitis, meningitis were reported in majority of the cases whereas, encephalitis and oophoritis were reported in a fewer subset of cases.
- Vaccination Status: A large proportion of mumps cases occurred in individuals who had received two or more doses of the MMR vaccine 57.4% had received two doses, and 21.3% had received three doses. In contrast, 8.5% of cases occurred among unvaccinated individuals and 6.4% among those who had received only one dose.

Further details are presented in Table 3.

**Table 3:** Descriptive characteristics of the included studies in this systematic review (*N* = 47).

Study characteristics	*n* (%) *
Eligible publications/outbreaks, *n*	47
Total individuals included from eligible studies, *n*	71,174
WHO region	
Africa (AFR)	0 (0.0%)
America (AMR)	12 (25.5%)
Eastern Mediterranean (EMR)	4 (8.5%)
Europe (EUR)	21 (44.7%)
South-East Asia (SEAR)	6 (12.8%)
Western Pacific (WPR)	4 (8.5%)
Year of outbreak, *n* (%) *	
2004–2009	18 (38.3%)
2010–2015	13 (27.7%)
2016–2020	16 (34.0%)
Study design, *n* (%) *	
Case control study	2 (4.2%)
Cohort study	2 (4.2%)
Cross sectional study	43 (91.6%)
Diagnostic test used, *n* (%) *	
RT-PCR	4 (8.5%)
Serological testing	3 (6.4%)
Clinical diagnosis	5 (10.6%)
Genotyping	2 (4.3%)
Combination of tests	31 (65.9%)
No diagnostic tests mentioned	2 (4.3%)
Population characteristics	
Age group, *n* (%) *	
0–10 years	32 (66.7%)
11–18 years	12 (25.0%)
≥19 years	4 (8.3%)
Gender, *n* (%) *	
Both male and female	43 (91.5%)
Missing baseline data for gender	4 (8.5%)
Vaccination Status, *n* (%) *	
Unvaccinated	4 (8.5%)
Only 1st dose	3 (6.4%)
Both 1st and 2nd dose	27 (57.4%)
All 3 doses	10 (21.3%)
Missing baseline data for vaccination	3 (6.4%)
*Column percentage	

### Complication rates of mumps outbreaks

**Figure 2:**
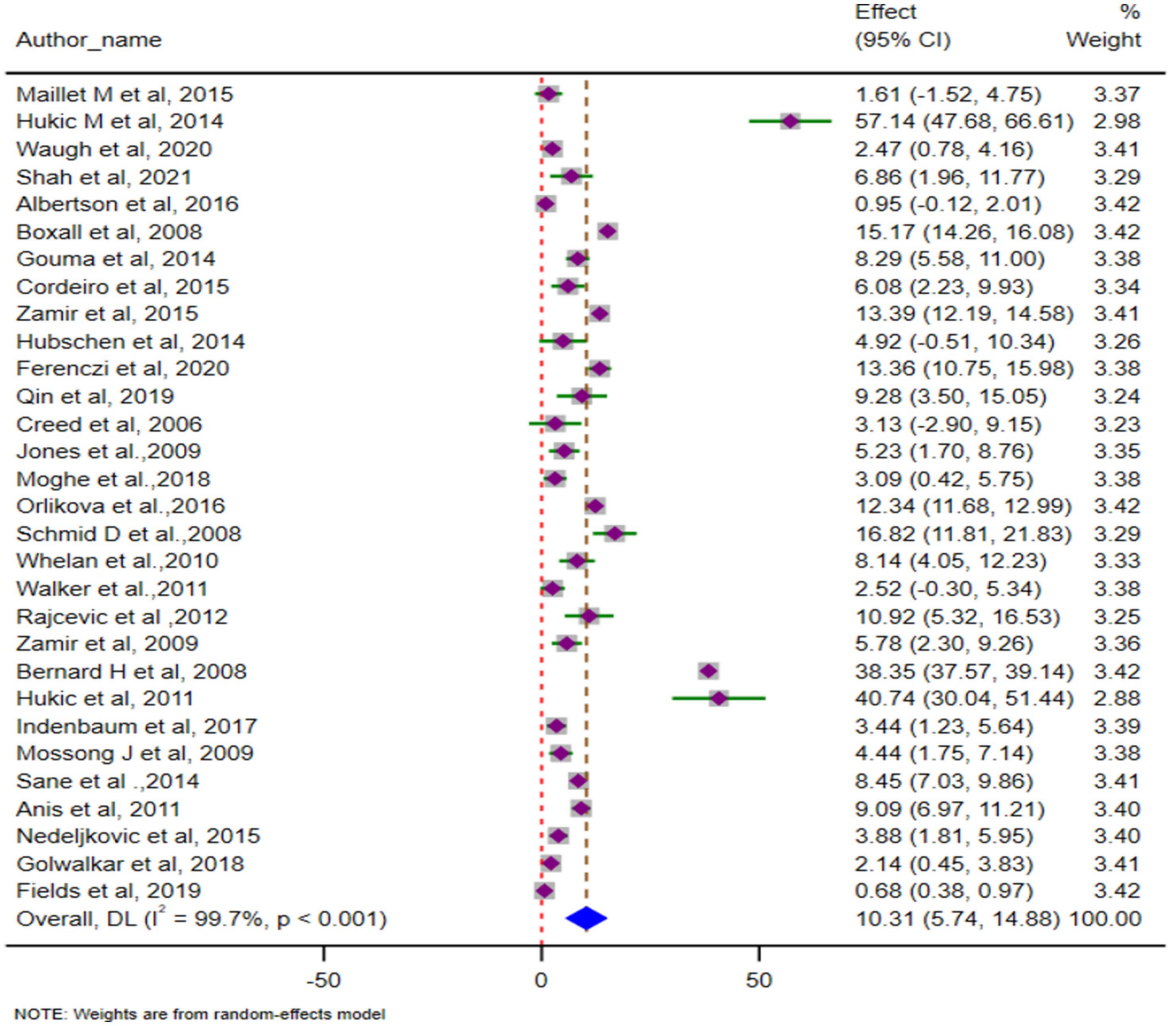
Pooled complication rate for mumps outbreaks globally (*N* = 30).

The pooled complication rate was 10.3% (95% CI: 5.7–14.9), with substantial heterogeneity (*p* < 0.001; *I*^2^ = 99.7%). A forest plot displaying the pooled complication rate is shown in Figure 2. Orchitis was the most common complication, with a pooled prevalence of 63.1% (95% CI: 49.8–76.6) across 22 studies. Other complications included encephalitis (0.2, 95% CI: 0.1–0.4), oophoritis, and hearing loss, which were infrequently reported. The full breakdown of complication rates is shown in Table 4.

**Table 4:** Subgroup analysis by complication rate of mumps outbreak globally.

Subgroup	Number of studies	Total Sample size	Complication rate (95% CI)	Heterogeneity statistics		
				Cochrane-Q	*I* ^2^	*p* value
By complications						
Orchitis	22	48,149	63.1 (49.8–76.6)	1243.9	100.0%	<0.001
Encephalitis	7	29,441	0.2 (0.1–0.4)	16.5	63.6%	0.011
Meningitis	15	43,819	1.4 (0.8–2.0)	161.0	91.3%	<0.001
Pancreatitis	11	30,096	0.8 (0.4–1.3)	174.5	94.3%	<0.001
Oophoritis	3	15,809	0.0 (−0.0–0.0)	2.6	24.1%	0.268
Hearing loss	2	5,563	0.3 (−0.0–0.6)	0.0	0.0%	0.947

### ARs of mumps outbreaks globally

**Figure 3:**
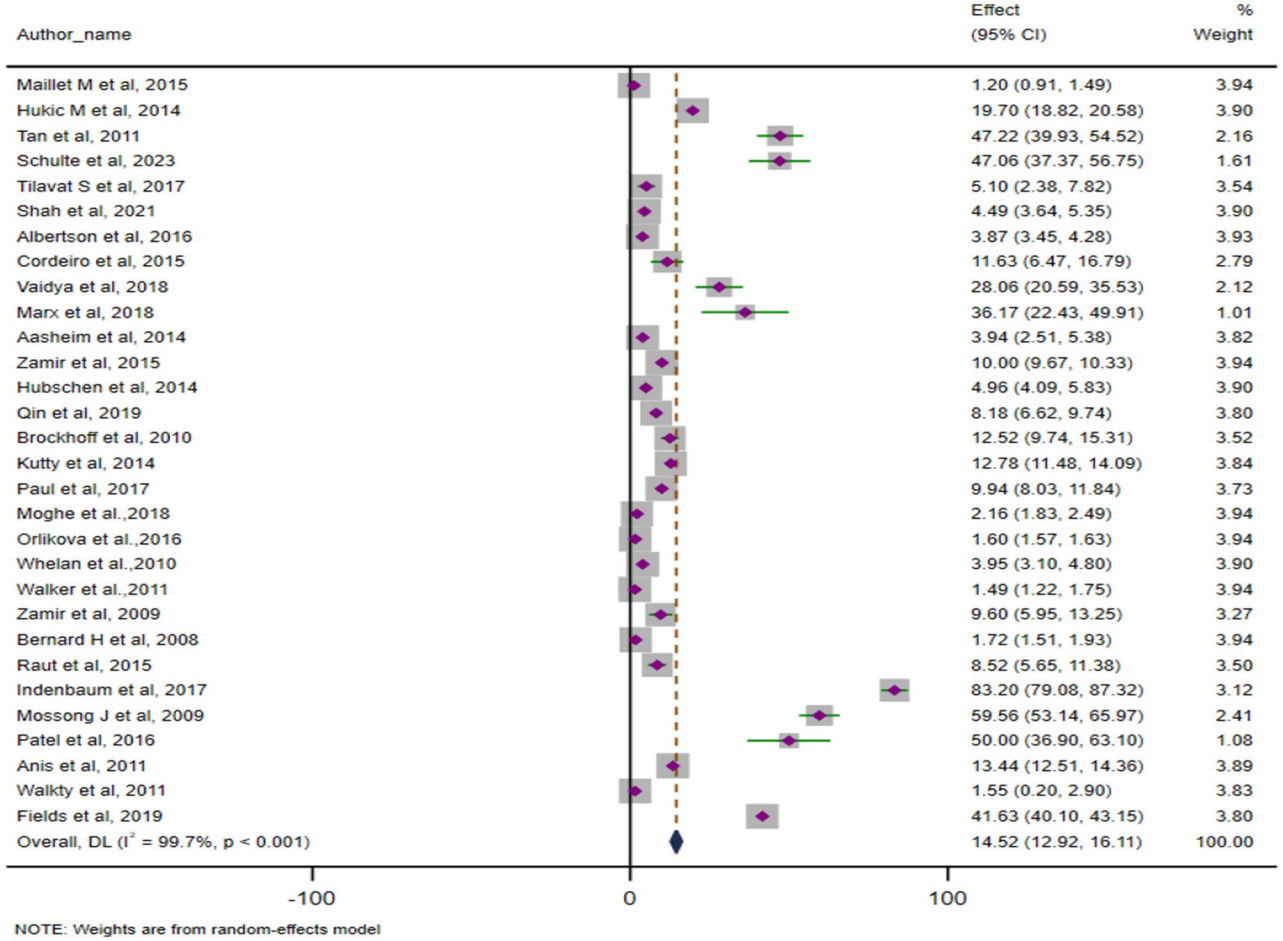
Pooled ARs for mumps outbreak globally (*N* = 30).

**Figure 4:**
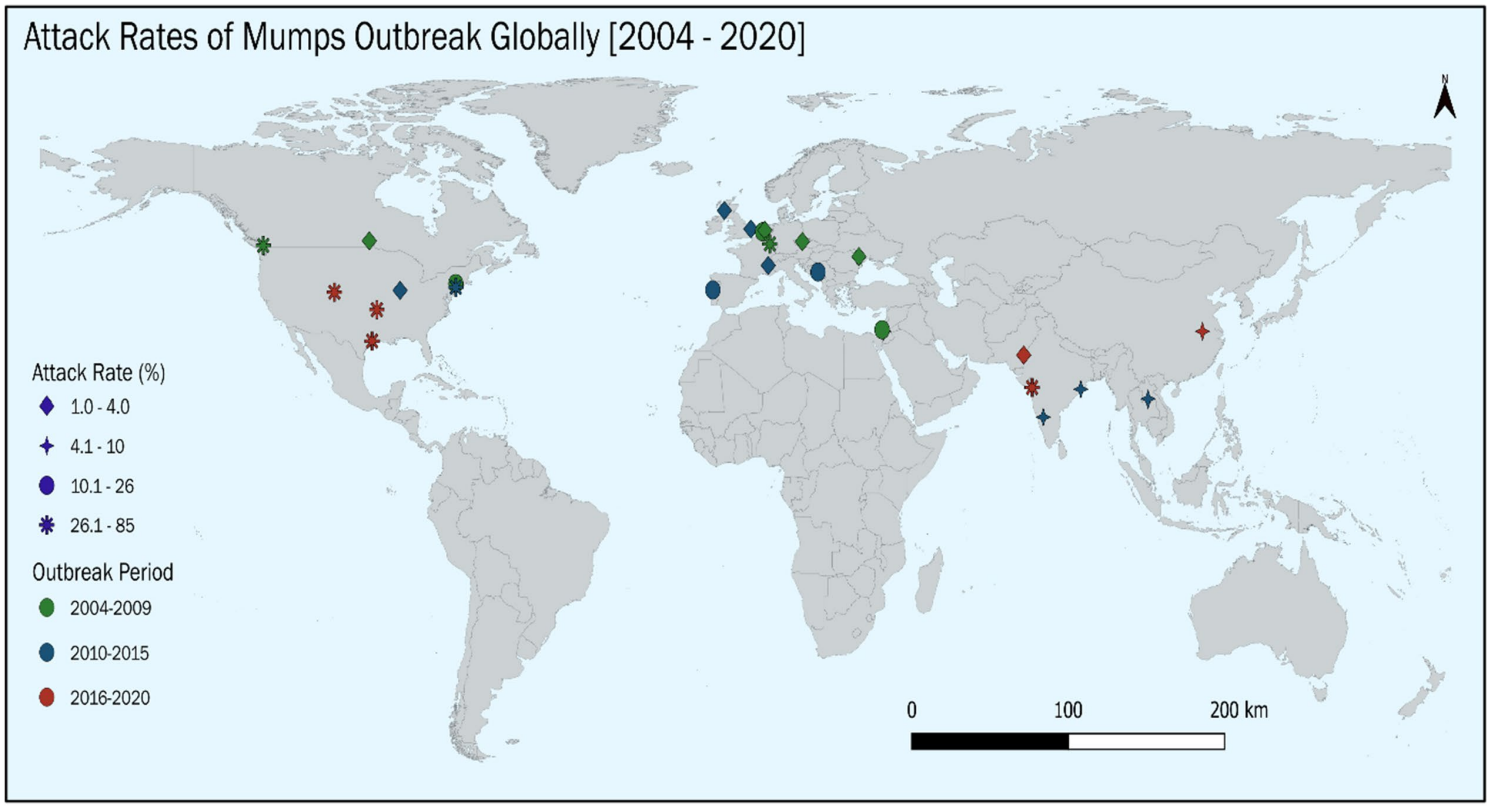
Global trend of mumps outbreak from the studies published between 2004 and 2024.

The pooled AR, based on 30 studies involving 50,643 cases, was 14.52% (95% CI: 12.91–16.11). Heterogeneity was significant (Cochrane Q = 10,238.2, *p* < 0.001; *I*^2^ = 99.7%). The forest plot of pooled ARs is shown in Figure 3. We have also depicted the global trend of mumps outbreak from the year 2004 to 2024. It is visually depicted in Figure 4.

### Subgroup analysis for AR determinants

Subgroup analysis for determinants of AR shows that adults (≥19 years) had the highest AR (31.8, 95% CI: 4.6–68.1, *I*^2^ = 0.0%), while children aged 0–10 years had an AR of 13.6% (95% CI: 11.0–16.0, *I*^2^ = 99.8%). AMR (29.2, 95% CI: 18.0–40.3, *I*^2^ = 99.7%) and EMR (28.8, 95% CI: 17.1–40.4, *I*^2^ = 99.8%) reported the highest ARs, while EUR (7.6%) and WPR (8.2%) exhibited lower rates with significant heterogeneity. Outbreaks reported between 2016 and 2020 showed a higher AR (28.0, 95% CI: 17.5–38.5) compared to earlier periods (8.5–12.6%). Individuals with only one vaccine dose had the highest AR (35.7, 95% CI: 8.7–62.5), while those with three doses showed significantly lower rates (10.1, 95% CI: 7.3–12.9). The detailed description is given in Table 5.

**Table 5:** Subgroup analysis for ARs determinants of mumps outbreaks globally.

Subgroup	Number of studies	Total Sample size	Attack rate (95% CI)	Heterogeneity statistics		
				Cochrane-Q	*I* ^2^	*p* value
By age group						
0–10 years	20	32,950	13.6 (11.0–16.0)	9423.8	99.8%	<0.001
11–18 years	7	17,089	11.7 (9.4–14.0)	574.5	98.8%	<0.001
≥19 years	3	604	31.8 (−4.6–68.1)	223.4	0.0%	<0.001
By region						
America (AMR)	8	6,481	29.2 (18.0–40.3)	2568.5	99.7%	<0.001
Eastern Mediterranean (EMR)	4	8,804	28.8 (17.1–40.4)	1239.0	99.8%	<0.001
Europe (EUR)	11	34,704	7.6 (6.1–9.0)	2111.7	99.5%	<0.001
South-East Asia (SEAR)	5	439	9.6 (4.5–14.7)	127.9	96.9%	<0.001
Western Pacific (WPR)	1	97	8.2 (6.6–9.7)	0.0	0.0%	<0.001
By year of outbreak						
2004–2009	11	37,897	12.6 (10.1–15.0)	3896.3	99.7%	<0.001
2010–2015	10	8,868	8.5 (5.6–11.4)	1814.5	99.5%	<0.001
2016–2020	9	3,878	28.0 (17.5–38.5)	4016.6	99.8%	<0.001
By vaccination status						
Unvaccinated	4	313	10.0 (5.5–14.5)	43.2	93.1%	<0.001
Only 1st dose	3	5,521	35.7 (8.7–62.5)	125.6	98.4%	<0.001
Both 1st and 2nd dose	14	31,541	16.4 (13.0–20.0)	7458.0	99.8%	<0.001
All 3 doses	5	10,474	10.1 (7.3–12.9)	522.9	99.2%	<0.001

### Association between various covariates with AR

A random-effects meta-regression with restricted maximum likelihood (REML) method was conducted to investigate the influence of study-level predictors on AR across 30 studies. The results indicated substantial residual heterogeneity, with a tau-squared of 2.50 and I^2^ of 99.80%. These metrics suggest that nearly all the variability in effect sizes across studies was due to true heterogeneity rather than sampling error. Despite this, the meta-regression model explained 0.00% of the between-study variance (*R*^2^ = 0.00%) and was not statistically significant overall (*p* = 0.58). None of the included covariates showed a statistically significant association with the AR. The detail description is provided in the Table 6.

**Table 6:** Multivariable meta-regression analysis for assessing the association between AR and various covariates.

Covariate	Coefficient	Standard error	*p* value	95% CI
Duration of outbreak (in months)	−0.02	0.02	0.35	−0.07 to 0.02
Country category	0.03	0.40	0.93	−0.75 to 0.82
Diagnostic test type	0.29	0.18	0.10	−0.06 to 0.64
Vaccination status	−0.08	0.36	0.81	−0.80 to 0.63
Age group	0.19	0.49	0.70	−0.79 to 1.17

### Leave-one-out sensitivity analysis

To assess the influence of individual studies on the overall pooled AR and complication rate leave-one-out sensitivity analysis was performed. The sensitivity analysis demonstrated the robustness of the finding, showing that no single study had a strong influence on the pooled estimate of AR and complication rate when results were computed excluding one study at time. The pooled AR remained stable, ranging from 12.03% (when Indenbaum et al., was removed) (16) to 16.24% (when Orlikova et al., was removed) (83), confirming the reliability of the overall result. However, the pooled complication rate also remains stable ranging from 8.76% (when Bernard H et al., is removed) (89) to a high of 10.66% (when Albertson et al., is removed) (66). The detailed description for both this estimate is provided in the Table 7.

**Table 7:** Influence of individual studies on pooled AR and complication rate as determined by leave-one-out sensitivity analysis.

Influence of individual studies on pooled ARs				
SN	Study omitted	Pooled estimates	Lower limit of 95% CI	Upper limit of 95% CI
1	Maillet et al. (59)	15.34	13.60	17.08
2	Hukic et al. (60)	13.99	12.47	15.51
3	Tan et al. (61)	13.77	12.16	15.37
4	Schulte et al. (62)	13.97	12.37	15.58
5	Tilavat et al. (64)	14.86	13.24	16.49
6	Shah et al. (65)	14.95	13.31	16.59
7	Albertson et al. (66)	15.07	13.40	16.75
8	Cordeiro et al. (69)	14.60	12.98	16.22
9	Vaidya et al. (70)	14.22	12.60	15.83
10	Marx et al. (71)	14.29	12.69	15.90
11	Aasheim et al. (72)	14.95	13.31	16.58
12	Zamir et al. (73)	14.39	12.87	15.92
13	Hubschen et al. (74)	14.92	13.29	16.56
14	Qin et al. (76)	14.76	13.14	16.39
15	Brockhoff et al. (30)	14.58	12.96	16.20
16	Kutty et al. (78)	14.55	12.93	16.16
17	Paul et al. (79)	14.69	13.06	16.31
18	Moghe et al. (82)	15.24	13.53	16.96
19	Orlikova et al. (83)	16.24	14.03	18.45
20	Whelan et al. (85)	14.97	13.33	16.61
21	Walker et al. (86)	15.40	13.63	17.17
22	Zamir et al. (88)	14.68	13.06	16.31
23	Bernard et al. (89)	15.62	13.75	17.50
24	Raut et al. (91)	14.73	13.11	16.36
25	Indenbaum et al. (16)	12.03	10.52	13.54
26	Mossong et al. (92)	13.35	11.76	14.95
27	Patel et al. (93)	14.12	12.52	15.72
28	Anis et al. (12)	14.46	12.87	16.06
29	Walkty et al. (95)	15.05	13.41	16.68
30	Fields et al. (101)	12.82	11.40	14.25
	Combined	14.52	12.92	16.11
Influence of individual studies on pooled complication rates				
1	Maillet et al. (59)	10.62	5.95	15.28
2	Hukic et al. (60)	8.87	4.26	13.48
3	Waugh et al. (63)	10.60	5.88	15.31
4	Shah et al. (65)	10.43	5.77	15.08
5	Albertson et al. (66)	10.66	5.86	15.46
6	Boxall et al. (67)	10.16	5.34	14.97
7	Gouma et al. (68)	10.38	5.71	15.06
8	Cordeiro et al. (69)	10.46	5.80	15.12
9	Zamir et al. (73)	10.22	5.45	14.98
10	Hubschen et al. (74)	10.49	5,84	15.15
11	Ferenczi et al. (75)	10.21	5.53	14.88
12	Qin et al. (76)	10.35	5.69	15.0
13	Creed et al. (77)	10.55	5.90	15.20
14	Jones et al. (81)	10.49	5.82	15.15
15	Moghe et al. (82)	10.57	5.89	15.24
16	Orlikova et al. (83)	10.29	5.21	15.37
17	Schmid et al. (84)	10.09	5.44	14.74
18	Whelan et al. (85)	10.39	5.73	15.05
19	Walker et al. (86)	10.59	5.91	15.26
20	Rajcevic et al. (87)	10.29	5.64	14.94
21	Zamir et al. (88)	10.47	5.81	15.14
22	Bernard et al. (89)	8.76	6.17	11.35
23	Hukic et al. (90)	9.41	4.78	14.04
24	Indenbaum et al. (16)	10.56	5.87	15.25
25	Mossong et al. (92)	10.52	5.84	15.20
26	Sane et al. (94)	10.39	5.64	15.14
27	Anis et al. (12)	10.36	5.67	15.05
28	Nedeljkovic et al. (99)	10.54	5.85	15.24
29	Golwalker et al. (100)	10.61	5.89	15.32
30	Fields et al. (101)	10.65	5.99	15.32

### Quality assessment and publication bias

Quality assessment using JBI tools showed that 96% of studies were high quality (scores >70%) (95), with only two studies rated as having moderate risk (50–69%) (28, 57). No studies were excluded based on quality assessment. A detailed quality assessment is provided in Supplementary Table 5. Funnel plot analysis and Egger’s regression test showed minimal publication bias, with no significant asymmetry detected Supplementary Figure 1.

## Discussion

This systematic review and meta-analysis assessed mumps outbreaks globally over the last two decades (2004–2024). We included 47 studies with 71,174 cases from 21 countries. The pooled AR of mumps outbreaks was 14.5%, while the overall complication rate was 10.3%, with orchitis being the most common complication. Peaks in outbreak activity were observed during 2004–2009 (38.3%) and 2016–2020 (34.6%), with most outbreaks occurring in the EUR (44.7%) and AMR (25.5%). Children were the most affected demographic, constituting 66.7% of cases, and 57.4% of cases occurred in individuals who had received two doses of the MMR vaccine.

The pooled AR of 14.5% varied significantly across age groups, regions, and vaccination statuses. Adults exhibited the highest AR, likely due to waning immunity and reduced natural exposure to mumps. The AR in the AMR (29.2%) was significantly higher than in the EUR (7.6%) and SEAR (9.6%), reflecting disparities in vaccination schedules, outbreak settings, and demographic characteristics. Similar findings were reported in a U. S. study by Clemmons et al. (102), which documented comparable ARs during outbreaks. In contrast, studies from China (76) and the Netherlands (33) reported lower ARs, ranging from 8.2 to 9.5%, suggesting that regional variations in vaccination coverage, waning immunity, and population density influence outbreak dynamics (103). These findings highlight the importance of regional monitoring and tailoring vaccination strategies to local epidemiological contexts.

A critical finding of this review is the high proportion of mumps cases among vaccinated individuals, particularly those with two doses of the MMR vaccine (57.4%) which likely reflects the widespread adoption of the two-dose schedule in many countries. The AR was highest among individuals with a single dose (35.7%) and lowest among those with three doses (10.1%), demonstrating the protective effect of booster doses. Studies such as Nelson et al. (104) and Cardemil et al. (105) corroborate these results, showing that a third dose reduces ARs by 60 to 78% compared to two doses. Despite this, the resurgence of outbreaks among fully vaccinated individuals underscores challenges related to waning immunity and evolving viral strains. Evidence indicates that MMR vaccine effectiveness decreases substantially over time, from 82% within 5 years of a single dose to 41% after 10 years (106). Similar declines are seen with two doses, highlighting the necessity of booster doses, especially in high-risk populations (78, 107). The best possible reason for increased risk after a single dose among vaccinated individuals is the waning of immunity and variability of vaccine effectiveness depending upon the age of vaccination and the time since vaccination (9).

The pooled complication rate of 10.3% aligns with findings from similar settings, with orchitis being the most common complication (63.1%). Complications such as encephalitis and hearing loss were rare but remain significant due to their potential long-term impact. Studies from the United Kingdom (108) and Korea (109) reported complication rates ranging from 5.3 to 16%, with variability influenced by vaccination coverage and diagnostic practices. Orchitis, particularly in post-pubertal males, is a notable concern due to its association with testicular atrophy and infertility (110). These findings emphasize the critical role of vaccination in reducing the severity and prevalence of complications. However, the resurgence of outbreaks in highly vaccinated populations raises concerns about herd immunity, underscoring the need for booster doses to mitigate severe outcomes.

Temporal trends revealed cyclical surges in mumps outbreaks approximately every 5–10 years, consistent with previous studies (111, 112). The absence of published evidence of mumps outbreaks beyond 2020 in the studies included in this review could be attributed to several factors, particularly those associated with the global COVID-19 pandemic and its aftermath. During the COVID-19 pandemic, public health priorities shifted dramatically, focusing almost exclusively on managing SARS-CoV-2 transmission and its associated burden on healthcare systems. This shift likely diverted resources, attention, and surveillance capabilities away from other infectious diseases, including mumps. For instance, in 2021, the overall notification rate was 0.4 cases per 100,000 population by European Union Member States. This was substantially lower than the notification rates observed in the previous 4 years, which ranged from 1.7 to 4.2 cases per 100,000 (113). This decline may reflect underreporting rather than an actual reduction in mumps incidence, as healthcare systems were overwhelmed, and routine disease surveillance was disrupted. Although data beyond 2020 were not included in our meta-analysis, but the reemergence of cases with the relaxation of restrictions underscores the enduring challenges of mumps control. For example, the United States reported 328 cases in 2024, reflecting the resurgence of outbreaks post-pandemic (114).

The geographical analysis demonstrated that mumps outbreaks are truly global, with substantial variation across WHO regions (115). EUR and AMR reported the majority of outbreaks, reflecting robust surveillance systems and higher reporting rates (102, 116). In contrast, outbreaks in SEAR and the EMR were less frequently reported, potentially due to limited surveillance or underreporting (117–119). The absence of outbreak data from the African region more likely reflects the under-reporting and limitations in surveillance systems rather than the actual absence of outbreaks. A recent report has highlighted persistent gaps in vaccine coverage, high numbers of “zero-dose” children, and incomplete vaccine preventable disease surveillance in the African Region, all of which may contribute to under-ascertainment of mumps cases (120, 121). These findings underscore the need for strengthening surveillance systems and ensuring equitable vaccine access in resource-limited settings.

Global differences in the establishment and development of mumps vaccination programs have an impact on vaccine policy and outbreak trends. Due to declining immunity and decreased natural boosting, mumps outbreaks among vaccinated adolescents and young adults are becoming more frequent in nations with high incomes that include the US, Canada, Israel, and parts of Europe where two-dose MMR vaccination has been in place for decades. As a result, during outbreaks, a third MMR dose is recommended for high-risk groups (29, 105, 122). The introduction of a standard primary doses is a higher priority in LMICs, such as India, where the mumps vaccination is still not part of national schedules (123). After repeated outbreaks, China added a second dose in 2008 after initially implementing a one-dose schedule (76). These differences underscore that booster dose recommendations are context dependent.

According to recent data, the number of mumps cases worldwide has increased since the COVID-19 pandemic (124). Many vaccine-preventable diseases, including mumps, saw a significant decrease in incidence after 2020. This may be due to lack of reported data on mumps outbreaks after the year 2020 which may be explained by multiple factors related to COVID-19 pandemic. Firstly, non-pharmaceutical interventions (NPIs) implemented during this period to control and prevent the SARS-CoV-2 transmission, including the use of masks, school closures, and social distancing, these all factors substantially reduced the circulation of several respiratory and close-contact viruses, and likely suppressed mumps transmission as well (11, 125). Additionally, the challenges faced in routine childhood immunisation and health services during the COVID-19 pandemic have been documented globally, which may have altered both susceptibility and case detection (126). Finally, with delayed reporting and reduced notification of vaccine-preventable diseases during 2020–21 adversely affected the surveillance systems as well (127). Collectively, all these factors may have contributed to the observed decline in reported mumps outbreaks during this period. However, several nations have reported an upsurge in the incidence of mumps since these restrictions were relaxed. For instance, the incidence rate increased from 4.4 per million in 2021 to 11.3 per million in 2023 in the WHO European Region, and from 0.6 to 18.7 per million in Northeast Asia during the same time-period (124). These patterns resemble those seen in other diseases that can be prevented, like pertussis, and they point to a possible rebound effect brought on by immunity gaps that could have waned during the pandemic. This resurgence emphasises how crucial it is to maintain ongoing surveillance and have strong vaccination campaigns to stop outbreaks in the future.

The genetic evolution of the mumps virus, specifically the emergence and predominance of genotype G strains, has raised concerns about the effectiveness of existing vaccines derived from the Jeryl Lynn strain (genotype A) (128). While the Jeryl Lynn-based vaccine has significantly reduced mumps incidence, recent outbreaks in highly vaccinated populations suggest that antigenic differences between vaccine and circulating strains may compromise vaccine-induced immunity (128, 129). Studies have identified amino acid variations in key antigenic sites, such as the hemagglutinin-neuraminidase (HN) protein, between genotype A and genotype G strains, potentially affecting neutralizing antibody responses (130). In the Netherlands, outbreaks predominantly caused by genotype G have been documented among vaccinated individuals, indicating possible immune escape due to these antigenic differences (128).

Future research should focus on understanding the genetic evolution of mumps virus strains and its impact on vaccine efficacy. Longitudinal studies evaluating antibody persistence post-vaccination would provide crucial data for refining booster dose recommendations. Improved global surveillance systems and data-sharing initiatives are essential for strengthening outbreak preparedness and response. By integrating these strategies, public health systems can reduce the global burden of mumps, protect vulnerable populations, and improve overall immunization efforts.

This study has several strengths. As the first meta-analysis on mumps outbreaks in two decades, it includes data from diverse geographical regions, offering a global perspective on mumps epidemiology. The use of standardized inclusion criteria and rigorous quality assessment ensures the reliability of findings. However, there are limitations to consider. High heterogeneity was observed in many pooled estimates, likely due to variability in study designs, diagnostic methods, and reporting standards. The reliance on original studies may also have introduced publication bias, despite minimal asymmetry observed in funnel plots and Egger’s tests. During our regional analysis although our aim was to include data from the entire American region, only studies from the USA and Canada were identified and met the inclusion criteria. Therefore, the findings may not be generalizable to the entire region. Another potential limitation is the underreporting of the outbreak most commonly in low-resource settings for example from the African region. Weak surveillance infrastructure, limited laboratory capacity, and competing health priorities may have resulted in missed or unreported outbreaks, particularly in LMIC countries (131). Consequently, the global pooled estimates derived in this review may disproportionately reflect data from high- and middle-income countries with more robust surveillance systems, limiting their generalizability to low-resource settings.

## Conclusion

A pooled AR of 14.5% and a complication rate of 10.3% were observed in global mumps outbreaks over the past two decades, with adults exhibiting the highest AR of 31.8%. Booster doses significantly reduced ARs to 10.1%. Incorporating a third MMR booster dose into vaccination schedules should be considered, particularly for high-risk groups, to mitigate outbreak severity and reduce complications worldwide. Such recommendations are primarily applicable to countries with long-standing two-dose coverage and documented waning immunity such as US, Australia, Canada, Israel and Part of Europe. In LMIC countries that have not yet introduced mumps vaccination, such as India, priority should be given to implementing and strengthening routine two-dose immunization programs before considering booster strategies. While the pooled estimates provide important insights into the global burden of mumps outbreaks, the high heterogeneity observed across studies indicates that the results should be interpreted with caution. The robustness of the findings in sensitivity analyses suggests that they are not driven by any single study but highlight the urgent need for more standardized reporting and improved surveillance systems.

## Data Availability

The original contributions presented in the study are included in the article/Supplementary material, further inquiries can be directed to the corresponding author/s.
