# Supplementary material for: Epidemiological trends and determinants of mumps outbreaks: a systematic review and meta-analysis
Source: Front Public Health. 2025 Dec 4;13:1711759. doi: 10.3389/fpubh.2025.1711759 (PMC12711706; doi:10.3389/fpubh.2025.1711759)
Supplement: Supplementary file 2 [file Table_2.docx]

**Supplementary file S2: Search strategy for mumps outbreak**

| ***Sl.No.*** | ***Concepts*** | ***Search Strategy*** |
| --- | --- | --- |
| 01. | Mumps | ((((Mumps [MeSH Terms]) OR (Epidemic Parotitis [Title/Abstract])) OR (Paroti*[Title/Abstract])) OR (Mumps viruses [Title/Abstract])) OR (Myxovirus parotitidis[Title/Abstract]) |
| 02. | Outbreak | (((Disease Outbreaks [MeSH Terms]) OR (Disease hotspot [Title/Abstract])) OR (Outbreak*[Title/Abstract])) OR (Epidemics [Title/Abstract]) |
| 03 | Combining both | ((((Mumps[MeSH Terms]) OR (Epidemic Parotitis[Title/Abstract])) OR (Paroti*[Title/Abstract])) OR (Mumps viruses[Title/Abstract])) OR (Myxovirus parotitidis[Title/Abstract])  AND (((Disease Outbreaks[MeSH Terms]) OR (Disease hotspot[Title/Abstract])) OR (Outbreak*[Title/Abstract])) OR (Epidemics[Title/Abstract])))) |
