# Supplementary material for: Epidemiological trends and determinants of mumps outbreaks: a systematic review and meta-analysis
Source: Front Public Health. 2025 Dec 4;13:1711759. doi: 10.3389/fpubh.2025.1711759 (PMC12711706; doi:10.3389/fpubh.2025.1711759)
Supplement: Supplementary file 3 [file Table_3.docx]

**Supplementary Table 3: List of studies excluded from systematic review with reasons**

| **Study Number** | **Author(s), Study year** | **Included/Excluded** | **Reason for Exclusion** |
| --- | --- | --- | --- |
| 1 | Walker et al, 2021 | Included | NA |
| 2 | Takla et al, 2013 | Excluded | Excluded as ineligible as per inclusion criteria |
| 3 | Huang et al, 2009 | Excluded | Excluded as ineligible as per inclusion criteria |
| 4 | Maillet et al, 2015 | Included | NA |
| 5 | Farley et al, 1985 | Excluded | Excluded as ineligible as per inclusion criteria |
| 6 | Ginzailez et al, 2012 | Excluded | English study |
| 7 | Hukic et al, 2014 | Included | NA |
| 8 | Tan et al, 2011 | Included | NA |
| 9 | Schulte et al, 2023 | Included | NA |
| 10 | Waugh et al, 2020 | Included | NA |
| 11 | No authors | Excluded | Excluded as it is non original study |
| 12 | No authors | Excluded | Excluded as it is non original study |
| 13 | Shah et al, 2021 | Included | NA |
| 14 | Barskey et al, 2009 | Excluded | Excluded as it is non original study |
| 15 | Tilavat et al, 2017 | Included | NA |
| 16 | Albertson et al, 2016 | Included | NA |
| 17 | Boxall et al, 2008 | Included | NA |
| 18 | Gouma et al, 2014 | Included | NA |
| 19 | Cordeiro et al, 2015 | Included | NA |
| 20 | Ortuondo et al, 2007 | Excluded | Non english study |
| 21 | Vaidya et al, 2018 | Included | NA |
| 22 | Rodra et al, 1999 | Excluded | Excluded as ineligible as per inclusion criteria |
| 23 | Marx et al, 2018 | Included | NA |
| 24 | Aasheim et al, 2014 | Included | NA |
| 25 | Livingston et al, 2014 | Excluded | Full text not available |
| 26 | No authors | Excluded | Excluded as it is non original study |
| 27 | Zamir et al, 2015 | Included | NA |
| 28 | Sullivan et al, 1985 | Excluded | Excluded as ineligible as per inclusion criteria |
| 29 | Casella et al, 1997 | Excluded | Excluded as ineligible as per inclusion criteria |
| 30 | Hubschen et al, 2013 | Included | NA |
| 31 | Ferenczi et al, 2020 | Included | NA |
| 32 | Qin et al, 2019 | Included | NA |
| 33 | No authors | Excluded | Excluded as it is non original study |
| 34 | Creed et al, 2006 | Included | NA |
| 35 | Brockhoff et al, 2010 | Included | NA |
| 36 | Reaney et al, 2001 | Excluded | Excluded as it is a review article |
| 37 | Karagiannis et al, 2008 | Excluded | Excluded as it is non original study |
| 38 | Kutty et al, 2014 | Included | NA |
| 39 | Paul et al, 2017 | Included | NA |
| 40 | No authors | Excluded | Excluded as it is non original study |
| 41 | Cohen et al, 2007 | Included | NA |
| 42 | Jones et al, 2009 | Included | NA |
| 43 | No authors | Excluded | Excluded as it is non original study |
| 44 | Saha et al, 2012 | Excluded | Excluded as ineligible as per inclusion criteria |
| 45 | Sartorius et al, 2005 | Excluded | Full text not available |
| 46 | Oda et al, 1996 | Excluded | Excluded as ineligible as per inclusion criteria |
| 47 | Wharton et al, 1988 | Excluded | Excluded as ineligible as per inclusion criteria |
| 48 | Hersh et al, 1991 | Excluded | Excluded as ineligible as per inclusion criteria |
| 49 | Germann et al, 1996 | Excluded | Excluded as ineligible as per inclusion criteria |
| 50 | Orlikova et al, 2015 | Included | NA |
| 51 | Schmid et al, 2008 | Included | NA |
| 52 | Whelan et al, 2010 | Included | NA |
| 53 | Walker et al, 2011 | Included | NA |
| 54 | Lee et al, 2004 | Excluded | Excluded as ineligible as per inclusion criteria |
| 55 | Montes et al, 2002 | Excluded | Excluded as ineligible as per inclusion criteria |
| 56 | Vandermeulen et al, 2004 | Excluded | Full text not available |
| 57 | Rajcevic et al, 2012 | Included | NA |
| 58 | Zamir et al, 2009 | Included | NA |
| 59 | Roberts et al, 2009 | Excluded | Excluded as ineligible as per inclusion criteria |
| 60 | Kuhlman et al, 1994 | Excluded | Excluded as it is a systematic review article |
| 61 | Paccaud et al, 1995 | Excluded | Non english study |
| 62 | Henderson et al, 1952 | Excluded | Excluded as ineligible as per inclusion criteria |
| 63 | Bernard et al, 2008 | Included | NA |
| 64 | Pugh et al, 2002 | Excluded | Full text not available |
| 65 | Hukic et al, 2011 | Included | NA |
| 66 | Raut et al, 2015 | Included | NA |
| 67 | Indenbaum et al, 2017 | Included | NA |
| 68 | Mossong et al, 2009 | Included | NA |
| 69 | Patel et al, 2017 | Included | NA |
| 70 | Sane et al, 2014 | Included | NA |
| 71 | Araga et al, 2020 | Excluded | Non english study |
| 72 | Labuda et al, 2019 | Excluded | Reported with other condition |
| 73 | Anis et al, 2012 | Included | NA |
| 74 | Walkty et al, 2011 | Included | NA |
| 75 | Saboui et al, 2020 | Included | NA |
| 76 | Mckay et al, 2019 | Included | NA |
| 77 | Moghe et al, 2019 | Included | NA |
| 78 | No authors | Excluded | Excluded as it is non original study |
| 79 | Tiffany et al, 2018 | Included | NA |
| 80 | Baum et al, 2017 | Excluded | Lack of parametrical estimates |
| 81 | Dyer et al, 2017 | Excluded | Lack of parametrical estimates |
| 82 | Seaux et al, 2014 | Excluded | Lack of parametrical estimates |
| 83 | Baum et al, 2016 | Excluded | Lack of parametrical estimates |
| 84 | Nedeljkovic et al, 2015 | Included | NA |
| 85 | Bernstein et al, 2008 | Excluded | Lack of parametrical estimates |
| 86 | Golwalkar et al, 2018 | Included | NA |
| 87 | Fields et al, 2019 | Included | NA |
