# Supplementary material for: Epidemiological trends and determinants of mumps outbreaks: a systematic review and meta-analysis
Source: Front Public Health. 2025 Dec 4;13:1711759. doi: 10.3389/fpubh.2025.1711759 (PMC12711706; doi:10.3389/fpubh.2025.1711759)
Supplement: Supplementary file 4 [file Table_4.docx]

**Supplementary Table 4: Data extraction template for systematic literature review**

| Variable |  |
| --- | --- |
| Author |  |
| Year |  |
| Journal |  |
| Country |  |
| WHO Region |  |
| Duration of study |  |
| Time period of outbreak |  |
| Total Number of outbreaks |  |
| Mode of spread |  |
| Incubation period |  |
| Reporting of index case |  |
| Study setting |  |
| Study design |  |
| Age group |  |
| Mean age |  |
| Median age |  |
| Total males |  |
| Total females |  |
| Total sample size |  |
| Diagnostic test used |  |
| Case definition |  |
| Case definition (Suspected/ Probable/ Confirmed) |  |
| Case definition (Lab/ Clinical/ Epidemiological based) |  |
| Number of cases notified |  |
| Number of cases confirmed |  |
| Mean duration of illness |  |
| Clinical outcomes |  |
| Clinical features |  |
| Vaccination status of cases |  |
| Total number of unvaccinated cases |  |
| Total number of cases vaccinated with one dose |  |
| Total number of cases vaccinated with two doses |  |
| Total number of cases vaccinated with three doses |  |
| Mean length gap between vaccination and outbreak |  |
| Attack rate |  |
| Total population at risk |  |
| Outbreak management |  |
| Outbreak intervention |  |
| Total mortality |  |
| Total complications |  |
| Additional information’s |  |
| Number of cases with fever |  |
| Number of cases with cold and cough |  |
| Number of cases with difficulty in swallowing |  |
| Number of cases with earache |  |
| Number of cases with orchitis |  |
| Number of cases with encephalitis |  |
| Number of cases with meningitis |  |
| Number of cases with pancreatitis |  |
| Number of cases with mastitis |  |
| Number of cases with oophoritis |  |
| Number of cases with nephritis |  |
| Number of cases with hearing loss |  |
| Number of cases with groin pain |  |
