# Supplementary material for: Epidemiological trends and determinants of mumps outbreaks: a systematic review and meta-analysis
Source: Front Public Health. 2025 Dec 4;13:1711759. doi: 10.3389/fpubh.2025.1711759 (PMC12711706; doi:10.3389/fpubh.2025.1711759)
Supplement: Supplementary file 5 [file Table_5.docx]

**Supplementary Table 5**

**Quality assessment of cross-sectional studies using JBI checklist**

| **SN** | **Author Name, Year of publication** | **Was the sample frame appropriate to address the target population?** | **Were study participants sampled in an appropriate way?** | **Was the sample size adequate?** | **Were the study subjects and the setting described in detail?** | **Was the data analysis conducted with sufficient coverage of the identified sample?** | **Were valid methods used for the identification of the condition?** | **Was the condition measured in a standard, reliable way for all participants?** | **Was there appropriate statistical analysis?** | **Was the response rate adequate, and if not, was the low response rate managed appropriately?** | **Total Scoring** | **Quality Assessment** | **Overall Appraisal** |
| --- | --- | --- | --- | --- | --- | --- | --- | --- | --- | --- | --- | --- | --- |
| 1 | Maillet et al, 2015 | 1.YES | 1.YES | 1.YES | 1.YES | 1.YES | 1.YES | 1.YES | 1.YES | 1.YES | 9 | High Quality | Include |
| 2 | Tan et al, 2011 | 1.YES | 1.YES | 1.YES | 1.YES | 1.YES | 1.YES | 1.YES | 1.YES | 4. NA | 8 | High Quality | Include |
| 3 | Schulte et al, 2023 | 1.YES | 1.YES | 1.YES | 1.YES | 1.YES | 1.YES | 1.YES | 1.YES | 4. NA | 8 | High Quality | Include |
| 4 | Waugh et al, 2020 | 1.YES | 1.YES | 1.YES | 1.YES | 1.YES | 1.YES | 1.YES | 1.YES | 1.YES | 9 | High Quality | Include |
| 5 | Tilavat et al, 2017 | 1.YES | 1.YES | 1.YES | 1.YES | 1.YES | 1.YES | 1.YES | 1.YES | 1.YES | 9 | High Quality | Include |
| 6 | Shah et al, 2021 | 1.YES | 1.YES | 1.YES | 1.YES | 1.YES | 1.YES | 1.YES | 1.YES | 1.YES | 9 | High Quality | Include |
| 7 | Albertson et al, 2016 | 1.YES | 1.YES | 1.YES | 1.YES | 1.YES | 1.YES | 1.YES | 1.YES | 1.YES | 9 | High Quality | Include |
| 8 | Boxall et al, 2008 | 1.YES | 1.YES | 1.YES | 1.YES | 1.YES | 1.YES | 1.YES | 1.YES | 4. NA | 8 | High Quality | Include |
| 9 | Gouma et al, 2014 | 1.YES | 1.YES | 1.YES | 1.YES | 1.YES | 1.YES | 1.YES | 1.YES | 4. NA | 8 | High Quality | Include |
| 10 | Cordeiro et al, 2015 | 1.YES | 1.YES | 1.YES | 1.YES | 1.YES | 1.YES | 1.YES | 1.YES | 4. NA | 9 | High Quality | Include |
| 11 | Vaidya et al, 2018 | 1.YES | 1.YES | 1.YES | 1.YES | 1.YES | 1.YES | 1.YES | 1.YES | 4. NA | 9 | High Quality | Include |
| 12 | Marx et al, 2018 | 1.YES | 1.YES | 1.YES | 1.YES | 1.YES | 1.YES | 1.YES | 1.YES | 1.YES | 9 | High Quality | Include |
| 13 | Aasheim et al, 2014 | 1.YES | 1.YES | 1.YES | 1.YES | 1.YES | 1.YES | 1.YES | 1.YES | 1.YES | 9 | High Quality | Include |
| 14 | Zamir et al, 2015 | 1.YES | 1.YES | 1.YES | 1.YES | 1.YES | 1.YES | 1.YES | 1.YES | 1.YES | 9 | High Quality | Include |
| 15 | Hubschen et al, 2013 | 1.YES | 1.YES | 1.YES | 1.YES | 1.YES | 1.YES | 1.YES | 1.YES | 1.YES | 9 | High Quality | Include |
| 16 | Ferenczi et al, 2020 | 1.YES | 1.YES | 1.YES | 1.YES | 1.YES | 1.YES | 1.YES | 1.YES | 3.UNCLEAR | 8 | High Quality | Include |
| 17 | Creed et al, 2016 | 1.YES | 1.YES | 1.YES | 1.YES | 1.YES | 1.YES | 1.YES | 3.UNCLEAR | 3.UNCLEAR | 7 | High Quality | Include |
| 18 | Kutty et al, 2014 | 1.YES | 1.YES | 1.YES | 1.YES | 1.YES | 1.YES | 1.YES | 1.YES | 1.YES | 9 | High Quality | Include |
| 19 | Paul et al, 2017 | 1.YES | 1.YES | 1.YES | 1.YES | 1.YES | 1.YES | 1.YES | 1.YES | 1.YES | 9 | High Quality | Include |
| 20 | Cohen et al, 2007 | 1.YES | 1.YES | 1.YES | 1.YES | 1.YES | 1.YES | 1.YES | 1.YES | 1.YES | 9 | High Quality | Include |
| 21 | Jones et al, 2009 | 1.YES | 1.YES | 1.YES | 1.YES | 1.YES | 1.YES | 1.YES | 1.YES | 4. NA | 8 | High Quality | Include |
| 22 | Moghe et al, 2018 | 1.YES | 1.YES | 1.YES | 1.YES | 1.YES | 1.YES | 1.YES | 1.YES | 4. NA | 8 | High Quality | Include |
| 23 | Orlikova et al, 2016 | 1.YES | 1.YES | 1.YES | 1.YES | 1.YES | 1.YES | 1.YES | 1.YES | 4. NA | 8 | High Quality | Include |
| 24 | Schmid et al, 2008 | 1.YES | 1.YES | 1.YES | 1.YES | 1.YES | 1.YES | 1.YES | 1.YES | 4. NA | 8 | High Quality | Include |
| 25 | Whelan et al, 2010 | 1.YES | 1.YES | 1.YES | 1.YES | 1.YES | 1.YES | 1.YES | 1.YES | 4. NA | 8 | High Quality | Include |
| 26 | Walker et al, 2011 | 1.YES | 1.YES | 1.YES | 1.YES | 1.YES | 1.YES | 1.YES | 1.YES | 4. NA | 8 | High Quality | Include |
| 27 | Rajcevic et al, 2012 | 1.YES | 1.YES | 1.YES | 1.YES | 1.YES | 1.YES | 1.YES | 1.YES | 4. NA | 8 | High Quality | Include |
| 28 | Zamir et al, 2009 | 1.YES | 1.YES | 1.YES | 1.YES | 1.YES | 1.YES | 1.YES | 1.YES | 4. NA | 8 | High Quality | Include |
| 29 | Bernard et al, 2008 | 1.YES | 1.YES | 1.YES | 1.YES | 1.YES | 1.YES | 1.YES | 1.YES | 4. NA | 8 | High Quality | Include |
| 30 | Hukic et al, 2011 | 1.YES | 1.YES | 1.YES | 1.YES | 1.YES | 1.YES | 1.YES | 1.YES | 3.UNCLEAR | 8 | High Quality | Include |
| 31 | Raut et al, 2015 | 1.YES | 1.YES | 1.YES | 1.YES | 1.YES | 1.YES | 1.YES | 1.YES | 4. NA | 8 | High Quality | Include |
| 32 | Indenbaum et al, 2017 | 1.YES | 1.YES | 1.YES | 1.YES | 1.YES | 1.YES | 1.YES | 1.YES | 4. NA | 8 | High Quality | Include |
| 33 | Mossong et al, 2009 | 1.YES | 1.YES | 1.YES | 1.YES | 1.YES | 1.YES | 1.YES | 1.YES | 1.YES | 9 | High Quality | Include |
| 34 | Patel et al, 2016 | 1.YES | 1.YES | 1.YES | 1.YES | 1.YES | 1.YES | 1.YES | 1.YES | 1.YES | 9 | High Quality | Include |
| 35 | Sane et al, 2014 | 1.YES | 1.YES | 1.YES | 1.YES | 1.YES | 1.YES | 1.YES | 1.YES | 1.YES | 9 | High Quality | Include |
| 36 | Anis et al, 2011 | 1.YES | 1.YES | 1.YES | 1.YES | 1.YES | 1.YES | 1.YES | 1.YES | 1.YES | 9 | High Quality | Include |
| 37 | Walkty et al, 2011 | 1.YES | 1.YES | 1.YES | 1.YES | 1.YES | 1.YES | 1.YES | 1.YES | 1.YES | 9 | High Quality | Include |
| 38 | Saboui et al, 2020 | 1.YES | 1.YES | 1.YES | 1.YES | 1.YES | 1.YES | 1.YES | 1.YES | 1.YES | 9 | High Quality | Include |
| 39 | McKay et al, 2019 | 1.YES | 1.YES | 1.YES | 1.YES | 1.YES | 1.YES | 1.YES | 1.YES | 1.YES | 9 | High Quality | Include |
| 40 | Tiffany et al, 2018 | 1.YES | 1.YES | 1.YES | 1.YES | 1.YES | 1.YES | 1.YES | 1.YES | 1.YES | 9 | High Quality | Include |
| 41 | Nedeljkovic et al, 2015 | 1.YES | 1.YES | 1.YES | 1.YES | 1.YES | 1.YES | 1.YES | 1.YES | 1.YES | 9 | High Quality | Include |
| 42 | Golwalkar et al, 2018 | 1.YES | 1.YES | 1.YES | 1.YES | 1.YES | 1.YES | 1.YES | 1.YES | 3.UNCLEAR | 8 | High Quality | Include |
| 43 | Fields et al, 2019 | 1.YES | 1.YES | 1.YES | 1.YES | 1.YES | 1.YES | 1.YES | 1.YES | 3.UNCLEAR | 8 | High Quality | Include |

**Quality assessment of cohort studies using JBI checklist**

| **Author Name, Year of publication** | **Were the two groups similar and recruited from the same population?** | **Were the exposures measured similarly to assign people to both exposed and unexposed groups?** | **Was the exposure measured in a valid and reliable way?** | **Were confounding factors identified?** | **Were strategies to deal with confounding factors stated?** | **Were the groups/participants free of the outcome at the start of the study (or now of exposure)?** | **Were the outcomes measured in a valid and reliable way?** | **Was the follow up time reported and sufficient to be long enough for outcomes to occur?** | **Was follow up complete, and if not, were the reasons to loss to follow up described and explored?** | **Were strategies to address incomplete follow up utilized?** | **Was appropriate statistical analysis used?** | **Total Scoring** | | **Quality Assessment** | **Overall Appraisal** |
| --- | --- | --- | --- | --- | --- | --- | --- | --- | --- | --- | --- | --- | --- | --- | --- |
| Hukic et al, 2014 | 1.YES | 3.UNCLEAR | 1.YES | 2. NO | 2. NO | 3.UNCLEAR | 1.YES | 1.YES | 3.UNCLEAR | 4.NA | 1.YES | 5 | Moderate quality | | Included |
| Brockhoff et al, 2010 | 1.YES | 3.UNCLEAR | 1.YES | 2. NO | 2. NO | 3.UNCLEAR | 1.YES | 1.YES | 3.UNCLEAR | 4.NA | 1.YES | 5 | Moderate quality | | Included |

**Quality assessment of case control studies using JBI checklist**

| **Author Name, Year of publication** | **Were the groups comparable other than the presence of disease in cases or the absence of disease in controls?** | **Were cases and controls matched appropriately?** | **Were the same criteria used for identification of cases and controls?** | **Was exposure measured in a standard, valid and reliable way?** | **Was exposure measured in the same way for cases and controls?** | **Were confounding factors identified?** | **Were strategies to deal with confounding factors stated?** | **Were outcomes assessed in a standard, valid and reliable way for cases and controls?** | **Was the exposure period of interest long enough to be meaningful?** | **Was appropriate statistical analysis used?** | **Total Scoring** | **Quality Assessment** | **Overall Appraisal** |
| --- | --- | --- | --- | --- | --- | --- | --- | --- | --- | --- | --- | --- | --- |
| Walker et al, 2021 | 1.YES | 1.YES | 1.YES | 1.YES | 1.YES | 1.YES | 1.YES | 1.YES | 1.YES | 1.YES | 10 | High Quality | Include |
| Qin W et al, 2019 | 1.YES | 1.YES | 1.YES | 1.YES | 1.YES | 1.YES | 1.YES | 1.YES | 1.YES | 1.YES | 10 | High Quality | Include |
